# Does Vancomycin Wrapping in Anterior Cruciate Ligament Reconstruction Affect Tenocyte Activity In Vitro?

**DOI:** 10.3390/antibiotics10091087

**Published:** 2021-09-08

**Authors:** Rocco Papalia, Claudia Cicione, Fabrizio Russo, Luca Ambrosio, Giuseppina Di Giacomo, Gianluca Vadalà, Vincenzo Denaro

**Affiliations:** Laboratory of Regenerative Orthopaedics, Department of Orthopaedic and Trauma Surgery, Campus BioMedico University of Rome, 00128 Rome, Italy; r.papalia@unicampus.it (R.P.); c.cicione@unicampus.it (C.C.); fabrizio.russo@unicampus.it (F.R.); l.ambrosio@unicampus.it (L.A.); g.digiacomo@unicampus.it (G.D.G.); denaro@unicampus.it (V.D.)

**Keywords:** anterior cruciate ligament, infection, tendon, vancomycin, human primary tenocytes, hamstring graft, septic arthritis

## Abstract

Knee septic arthritis is a devastating complication following anterior cruciate ligament (ACL) reconstruction. To prevent this issue, intraoperative soaking of ACL grafts with vancomycin is often performed before implantation. Although vancomycin cytotoxicity has been reported several times, little is known about its biological effect on tenocytes. The aim of this study was to evaluate the in vitro effects of vancomycin on human primary tenocytes (hTCs). hTCs were isolated from hamstring grafts of four patients undergoing ACL reconstruction. After expansion, hTCs were treated with different concentrations of vancomycin (0, 2.5, 5, 10, 25, 50 and 100 mg/mL) for 10, 15, 30 and 60 min. In vitro cytotoxicity was evaluated measuring metabolic activity, cell toxicity, and apoptosis. hTC metabolic activity was affected starting from 10 mg/mL vancomycin and decreased markedly at 100 mg/mL. Cell viability remained unaffected only at a concentration of 2.5 mg/mL vancomycin. Vancomycin cytotoxicity was detected from 10 mg/mL after 15 min and at all higher concentrations. Cells died when treated with concentrations higher than 5 mg/mL. The use of this antibiotic on tendons to prevent infections could be useful and safe for resident cells if used at a concentration of 2.5 mg/mL for up to 1 h of treatment.

## 1. Introduction

Anterior cruciate ligament (ACL) injury is among the most common and economically costly sport injuries, frequently requiring expensive surgery and rehabilitation [1]. It affects roughly 1 in 3000 people per year and an estimated 400,000–500,000 ACL reconstructions (ACLR) are performed annually worldwide [2,3]. Post-operative knee septic arthritis represents a serious complication with an incidence rate between 0.14 and 1.7% [4]. The main risk factors include revision surgery and the use of hamstring tendon autografts [5,6,7,8,9]. Several microorganisms have been isolated from the synovial fluid of patients affected by septic arthritis such as *Staphylococci*, the most prevalent coagulase-negative (CNS) bacteria, followed by *Staphylococcus aureus*, *Enterobacteriaceae*, *Propionibacterium acnes*, *Pseudomonas* spp., and many others [10,11,12,13]. Early diagnosis and immediate antibiotic treatment are crucial. However, the eradication of infections is challenging due to both the poor vascularization of the tendon and bacterial biofilm formation [14].

A common practice to avoid knee septic arthritis after ACLR is the “vancomycin wrap”, first described by Vertullo and colleagues [15], involving the soaking of the graft for 10–15 min within a sterile gauze swab previously saturated with 5 mg/mL vancomycin [16,17,18]. Vancomycin was first described in 1952 and approved by the FDA in 1958 as an antibiotic active against Gram-positive and penicillin-resistant *Staphylococci*. Vancomycin was rapidly eclipsed by equally effective and less toxic antibiotics. However, at the beginning of the 1980s, an increase in its use occurred due to its efficacy against resistant pathogens such as *Clostridium difficile*, *Streptococcus pneumoniae*, and methicillin-resistant *Staphylococcus aureus* (MRSA) [19]. The use of vancomycin in ACL wrap soaking was supported by its activity against the most common pathogens involved in septic arthritis and its low allergenicity. Even though several studies have documented vancomycin toxicity on different musculoskeletal tissues and cells at therapeutical doses, little is known about the biological effect of such antimicrobial use on tendon-derived cells.

The purpose of this study was to evaluate the toxicity of different concentrations of vancomycin on human primary tenocytes (hTCs) in vitro. We hypothesized that a prolonged exposure of hTCs to the antibiotic and/or the increase of vancomycin concentration would result in a reduction of cell viability and metabolic activity, which may negatively impact on graft integrity and the success of surgery.

## 2. Results

The hamstring specimens used for the study were harvested from four patients who underwent ACLR of the right knee. The subjects included were three males and one female, with a mean age of 36.25 ± 13.15 years, without concomitant comorbidities or pharmacological treatments (Table 1).

### 2.1. Cell Metabolic Activity

**Figure 1:**
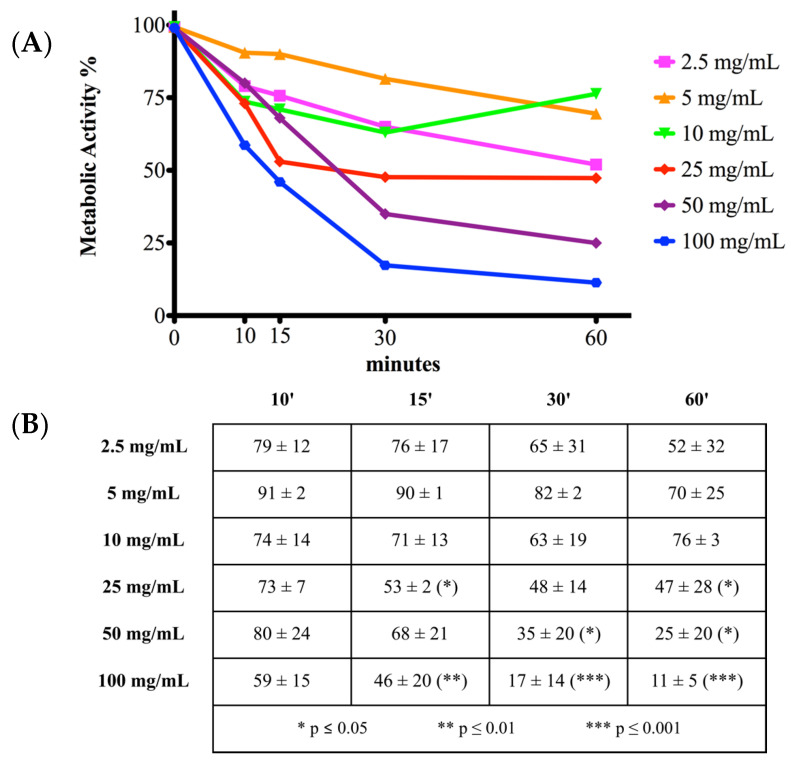
MTT dose-time curves. (**A**) Each point corresponds to a vancomycin treatment at a different concentration at a certain timepoint. Relative metabolic activity was calculated considering 0 mg/mL vancomycin as a baseline of 100%. (**B**) Relative metabolic activity was expressed as mean percentage ± standard deviation of at least three independent experiments. Mitochondrial activity was significantly reduced by 25 mg/mL vancomycin at 15 and 60 min, 50 mg/mL at 30 and 60 min, and 100 mg/mL at all timepoints after 10 min. Statistical significance was calculated versus the control group (0 mg/mL vancomycin) and reported as * *p* ≤ 0.05, ** *p* ≤ 0.01 and *** *p* ≤ 0.001. See text for details.

An indirect measurement of cell status is the analysis of mitochondrial or metabolic activity with MTT assay (Figure 1). Control cells were treated with 0 mg/mL of vancomycin and were assumed to have 100% mitochondrial activity. The results of vancomycin treatment at different concentrations were calculated using control cells as a reference. In the dose-time curves under study, lower concentrations of vancomycin (2.5, 5, and 10 mg/mL) reduced mitochondrial activity compared to 0 mg/mL without significant differences at all time points analyzed. After 15 min, mitochondrial activity reduced with 25 and 100 mg/mL vancomycin treatments by 53% ± 2% (*p* < 0.05) and 46% ± 20% (*p* < 0.01), respectively. One-hundred milligrams per milliliter of vancomycin significantly reduced cell activity at 30 min (17% ± 14%; *p* < 0.001) and at 60 min (11% ± 5%; *p* < 0.001). Finally, a significant detrimental effect of vancomycin was observed with 50 mg/mL after 60 min (25% ± 20%; *p* < 0.05).

### 2.2. Cell Viability and Toxicity

**Figure 2:**
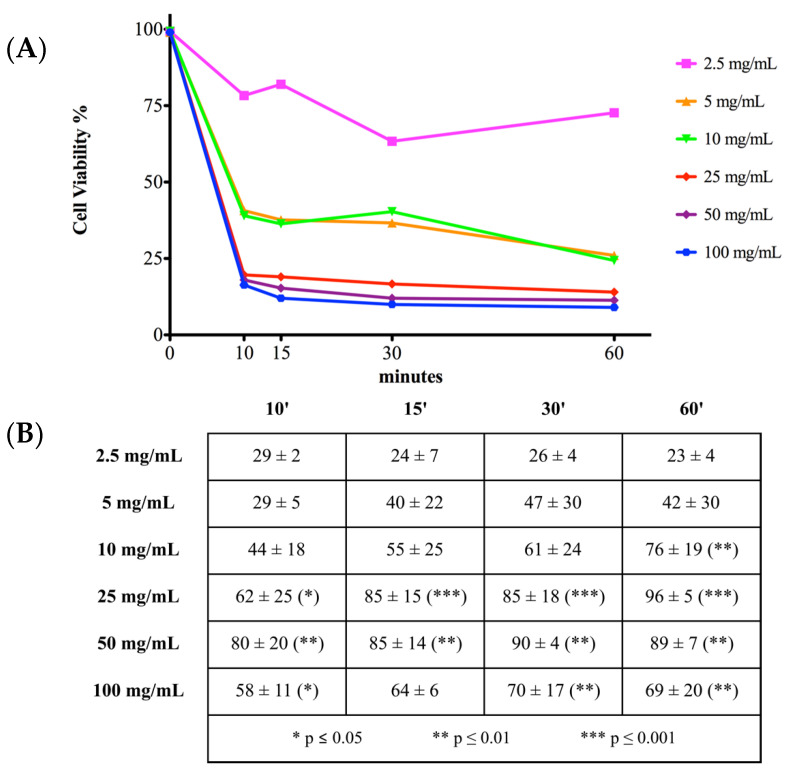
Cell viability dose-time curves. (**A**) Each point corresponds to a vancomycin treatment at a different concentration at a certain time point. Relative cell viability was calculated considering 0 mg/mL vancomycin as a baseline of 100%. (**B**) Cell viability results were expressed as mean reduction of cell viability ± standard deviation for at least three independent experiments. Cell viability was significantly reduced by 10 mg/mL vancomycin at 60 min as well as by 25 mg/mL, 50 mg/mL, and 100 mg/mL approximately at all timepoints. Statistical significance was calculated versus the control group (0 mg/mL vancomycin) and reported as * *p* ≤ 0.05, ** *p* ≤ 0.01 and *** *p* ≤ 0.001. See text for details.

**Figure 3:**
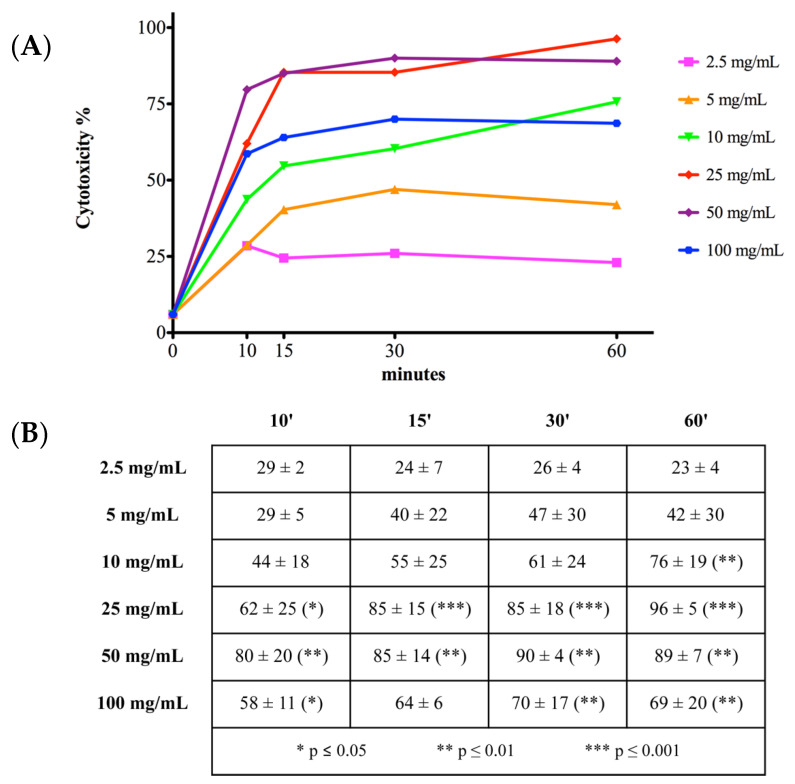
Cytotoxicity dose-time curves. (**A**) Each point corresponds to a vancomycin treatment at a different concentration at a certain time point. Relative cytotoxicity was calculated considering cells treated with MeOH for 30′ as a baseline of 100%. (**B**) Cytotoxicity results were expressed as mean percentage ± standard deviation for at least three independent experiments. Cytotoxicity was significantly increased by 10 mg/mL vancomycin at 60 min as well as by 25 mg/mL, 50 mg/mL, and 100 mg/mL approximately at all timepoints. Statistical significance was calculated versus the control group (0 mg/mL vancomycin) and reported as * *p* ≤ 0.05, ** *p* ≤ 0.01 and *** *p* ≤ 0.001. See text for details.

Membrane integrity was evaluated by live/dead assay to determine vancomycin viability and toxicity (Figure 2 and Figure 3). Vancomycin treatment at 0 mg/mL was considered as a control and assumed to correspond to 100% cell viability. The results of the other vancomycin concentrations were compared to the control. In contrast, cells treated with MeOH for 30 min were assumed to suffer 100% cell death and all the other treatments were calculated comparing each measurement starting from this reference. Cells treated with 2.5 mg/mL vancomycin showed a significant reduction of viability (Figure 2) after 30 min of treatment (54% ± 17%; *p* < 0.01) but did not show other significant alterations in viability and toxicity (Figure 2 and Figure 3) compared to 0 mg/mL. Cell viability for all the other concentrations of vancomycin were significantly lower compared to 0 mg/mL at any time point, ranging from 41% to 9%, as shown in the table (Figure 2, *p* < 0.001).

Regarding toxicity (Figure 3), the increase in cell death started to be significant after 60 min of treatment with 10 mg/mL vancomycin (76% ± 19%; *p* < 0.01) but was not significant with 2.5, 5, and 10 mg/mL at the other timepoints analyzed. Higher concentrations of vancomycin were cytotoxic at all the time points examined in the study. The calculated half maximal inhibitory concentration (IC50) was 3.033 to 9.286 mg/mL at 10 min, 2.845 to 9.228 mg/mL at 15 min, 1.497 to 6.879 mg/mL at 30 min, and 1.775 to 5.541 mg/mL at 60 min. No significant increase of cytotoxicity was found in hTCs at 0 mg/mL vancomycin compared to cells in culture medium at all timepoints (Figure S1).

### 2.3. Cell Apoptosis

To evaluate if toxic effects of higher concentrations of vancomycin caused cell death through apoptotic processes, Annexin V/PI flow cytometric assay was performed. Interestingly, hTCs were Annexin V-FITC/PI negative at all the concentrations analyzed, indicating the absence of early and late apoptosis. However, flow cytometric analysis after 24 h showed that cells treated with different concentrations of vancomycin at all time points recovered and were still negative for both Annexin V and PI (data not shown).

## 3. Discussion

The main findings of this study were that the dose and time of exposure to vancomycin, conventionally employed during ACL graft soaking, may significantly reduce hTC viability and metabolic activity in vitro.

Knee septic arthritis is one of the most feared and devastating complications following ACLR. Although relatively less common than other orthopaedic-related infections, the condition may require prolonged antibiotic treatments, multiple reoperations, cartilage loss, hardware and graft removal, and can lead to arthrofibrosis in the most severe cases [20]. Vancomycin is active against Gram-positive bacteria, typically infecting bacteria, has low allergenicity, and is less toxic to local tissues than alternative antibiotics, such as tobramycin, cefazolin, and gentamicin [21]. Vancomycin soaking of the graft is often performed during ACLR to reduce the risk of post-operative septic arthritis. Briefly, a surgical swab is imbibed in vancomycin (usually 5 mg/mL) and then wrapped around the graft for 10–15 min before implantation [15]. The effectiveness of this technique may be explained by the decontamination of a contaminated graft, which can occur during harvest preparation or passage through the portals in up to 22% of cases [22], by intra-articular elution of a loaded antibiotic reservoir, or both [13]. Indeed, after soaking, the graft itself may act as an antimicrobial reservoir capable of continuously eluting vancomycin over the minimum inhibitory concentration (MIC) needed to eliminate most microorganisms involved in post-ACLR septic arthritis (*S. aureus* = 0.25 mg/mL, *Streptococcus* = 0.25 mg/mL, *Enterococcus* spp. = 2 mg/mL) [23,24]. In an ex vivo study, Grayson et al. showed that vancomycin release from tendons started immediately after soaking and was maintained for at least 24 h, with a peak elution rate in the first 10 min, followed by a plateau at successive intervals. Furthermore, the elution rate was measurably increased when thicker tendons and higher vancomycin concentrations were employed, without ever reaching commonly accepted toxic concentrations [23]. The technique is easy to perform and has shown favorable results in terms of efficacy, safety, and cost-effectiveness [25]. Indeed, a recent meta-analysis by Naendrup et al. concluded that vancomycin-soaking of the graft dramatically reduced the rate of septic arthritis following ACLR, with no episodes of infection reported in the analyzed cohorts [26]. However, how long the graft should be presoaked and the biological effect of vancomycin concentration on graft tissues still remains unclear.

To our knowledge, this is the first study to evaluate dose-time effects of vancomycin on hTCs. In our experimental conditions, 2.5 mg/mL of vancomycin did not affect hTC viability up to 60 min with no increase in cell death, showing a behavior that did not significantly differ from soaking in saline solution. Conversely, 5 mg/mL, corresponding to the most used concentration for the vancomycin wrap procedure, resulted in a decrease in hTC viability of approximately 60% compared to cells in the control group after 10 min, which is the average duration of graft soaking [18,25,26]. The results obtained with live/dead assay and MTT assay indicated that, even at 2.5 ng/mL, cells reduced their metabolic activity by an average of 50% after 1 h of vancomycin treatment, while cell viability remained around 60% compared to 0 mg/mL at the same time point. While MTT is an indirect measure of cell viability and metabolic activity, the difference between these two assays could be due to a reduction in cell activity not directly correlated with the overall cell viability [27,28].

In the literature, several studies have reported the toxicity of vancomycin on different cell types and tissues. In vitro experiments on porcine chondrocytes showed that vancomycin exposure at 5 and 10 mg/mL for 1 h resulted in toxicity and caused cell death [29], while in vivo intra-articular injections of vancomycin reduced rabbit chondrocyte viability even at 1 mg/mL [30]. In contrast, 5 mg/mL vancomycin did not affect the cell number and alkaline phosphatase activity of human osteoblasts treated for 10 and 14 days [31], whereas 10 mg/mL was reported to cause death on MG-63 osteosarcoma cells in vitro [32]. Vancomycin had little effect on cell proliferation up to 1 mg/mL but could reduce osteogenic marker expression at 0.1 and 1 mg/mL in periosteal cells from rabbit tibia [33]. In another study, 125 mg/mL of vancomycin induced osteoblast toxicity and inhibited bone regeneration [21].

In a recent manuscript by Liu and colleagues, osteoblasts, myoblasts, and fibroblasts were cultured in the presence of different amounts of vancomycin (1, 3, 6, or 12 mg/cm^2^) for either 1 h or 48 h, in order to simulate the effects of a short (e.g., wound lavage) or a long (e.g., antibiotic-loaded spacers) exposure on cells participating in joint tissue repair. The authors found that 1 h vancomycin exposure reduced osteoblast and myoblast survival and migration only at the highest dosage (12 mg/cm^2^). Conversely, a prolonged vancomycin exposure significantly impaired both survival and migration in all cell types at all concentrations tested [34]. According to our results, it may be reasonable to reduce vancomycin concentration for graft soaking under 5 mg/mL to reduce the risk of graft damage without affecting antimicrobial efficacy, as the MIC for most bacteria involved in post-ACLR septic arthritis is approximately 2 mg/mL [23]. Moreover, as exposure to 2.5 mg/mL vancomycin did not result in toxicity at any time point, prolonged graft soaking over the 10 min course, e.g., due to intraoperative complications, may be reasonably safe, while longer incubation at higher concentrations may further affect tendon cell metabolic activity and viability.

Graft toxicity and resulting tenocyte death may theoretically lead to premature biomechanical graft failure with the need for reoperation. In an ex vivo study, Schüttler et al. [35] contaminated porcine flexor digitorum profundus tendons with *Staphylococcus* epidermidis and then soaked the specimens with 1, 2.5, 5, and 10 mg/mL vancomycin for either 10 or 20 min. Residual bacterial contamination and maximum load and stiffness were then evaluated. While all specimens were still contaminated after 10 min, no signs of infection were observed in the groups treated with 5 and 10 mg/mL vancomycin for 20 min after one week of culture, while 42.9% of tendons treated with 2.5 mg/mL still showed bacterial contamination. However, the study may be biased by both the small sample size and the method of contamination, which is far from a clinical scenario. Surprisingly, no sign of biomechanical impairment was noted in all groups.

This study has some limitations. The main relates to the in vitro experimental procedure, which differs from the in vivo environment of tenocytes. Further experiments will be performed to evaluate vancomycin toxicity on ex vivo tendon explants. As vancomycin chondrotoxicity has been described as well, the combined effect of the antibiotic on both tenocytes and chondrocytes should be investigated to define its role on the main joint cell types. Moreover, it has been proposed that vancomycin could jeopardize the ligamentization process [32,36], which is characterized by the progressive replacement of tendon specific features of the implanted grafts with ligamentous properties. It is recognized that the combined intra-articular remodeling and ligamentization of the graft dictate the biomechanical function of ACL reconstruction. It has been demonstrated that low concentrations of vancomycin have no deleterious effects on tenocytes [23] nor risk of re-rupture [25]. The present in vitro study did not analyze the effect of vancomycin on the ligamentization process or biomechanical properties; therefore, further studies should be carried out to confirm the mid- and long-term safety of the use of vancomycin.

## 4. Materials and Methods

### 4.1. Isolation and Culture of Human Tendon-Derived Primary Cells

Human hamstring samples were harvested from surgical waste materials of 4 patients undergoing ACLR. All patients signed written informed consent and all the procedures were approved by our Institutional Ethical Committee (patient data can be found in Table 1). Tendons were collected as they arrived from the surgery room and processed the same day. Tissues were washed two times with phosphate-buffered saline (PBS), minced into small pieces, and digested with 0.5 mg/mL of collagenase type II (Worthington, Lakewood, NJ, USA) for 2 h at 37 °C in RPMI medium with 5% fetal bovine serum (FBS). The digested material was centrifuged; the pellet was placed in a Petri dish and cultured at 37 °C and 5% CO_2_ in RPMI with 10% FBS, 1% penicillin/streptomycin (P/S) and 1% L-Glut (Supplementary Figure S2). The medium was changed twice a week. Cells were passaged with 1× Trypsin/EDTA when they reached 80–90% confluence for at least 3 passages (P3). Each cell culture was derived from a single donor.

### 4.2. Vancomycin Treatment

At the third passage, hTCs were plated and the medium was removed after 24 h. Cells were then treated with vancomycin (Hikma Italia S.P.A., Pavia, Italy) in saline solution (0.9% NaCl, B. Braun, Melsungen, Germany) at 0, 2.5, 5, 10, 25, 50, and 100 mg/mL for 10, 15, 30, and 60 min. Each treatment was performed in triplicate. Cells with culture medium and 0 mg/mL were used as a control. Cells treated with 70% MeOH for 30 min were used as a positive control for cytotoxicity. At each time point, cell metabolic activity, viability, toxicity, and apoptosis were assessed as described above.

### 4.3. Cell Activity

Cell metabolic activity was measured with MTT assay (Sigma-Aldrich, St. Louis, MO, USA), according to the manufacturer’s instructions. At the third passage, the cells were plated in 96-multi-well plates (1.5 × 10^4^ cells/well) and treated as described above. At each time point, after vancomycin treatment, cells were incubated for 4 h with 10% MTT solution. Its reduction by mitochondrial dehydrogenases to purple formazan crystals was determined reading the absorbance at 550 nm (Tecan Infinite M200 PRO).

### 4.4. Cell Viability and Toxicity

Cell toxicity was determined analyzing the membrane integrity through the LIVE/DEAD™ cell viability assay (Thermo Fisher Scientific), following the manufacturer’s instructions. At P3, cells were plated in 96-multi-well plates (1.5 × 10^4^ cells/well) and treated as previously described. After the treatment, cells were incubated for 30 min with ethidium homodimer-2 and calcein acetoxymethylester (AM) at room temperature and washed with PBS 3 times. Green and red fluorescence were quantified reading the fluorescence at 645 nm and 530 nm (Tecan Infinite M200 PRO).

### 4.5. Cell Apoptosis

Cell apoptosis was determined using flow cytometry Annexin V Apoptosis kit-FITC (Novus Biologicals-BioTechne, Minneapolis, MN, USA). At P3, cells were plated in 24-multi-well plates (3 × 10^4^ cells/well) and treated as previously described. After treatment, cells were washed with PBS, detached with trypsin/EDTA, and recovered. After 2 washes with PBS, cells were incubated with Annexin V/PI dyes for 20 min at room temperature, according to the manufacturer’s instructions. Blocking buffer was added and cells were analyzed with CytoFlex (Beckman Coulter, Brea, CA, USA).

### 4.6. Statistical Analysis

Each experiment was repeated at least three times with cells from 4 different donors. Results are expressed as the mean ± standard deviation (SD). The statistical analysis of the results was performed using one-way analysis of variance (ANOVA) with Dunnett’s post-test comparing each value to 0 mg/mL treatment (Prism 5 GraphPad Software, La Jolla, CA, USA). Nonlinear regression was performed to calculate the IC50. Statistical significance was denoted by * *p* < 0.05, ** *p* < 0.01, *** *p* < 0.001. Error bars represent SD.

## 5. Conclusions

Pre-soaking of ACL grafts in vancomycin is an efficacious, cost-effective, and safe option to reduce the rate of post-ACLR septic arthritis. To our knowledge, this is the first study to evaluate the dose-time effects of vancomycin on hTCs in vitro. From the data presented, vancomycin was shown to be a safe treatment at 2.5 mg/mL up to 60 min, while higher concentrations commonly used during the vancomycin wrap procedure may harm the graft tenocyte viability and metabolic activity.

## Figures and Tables

**Table 1:** Demographic characteristics of patients included in the study.

Patient	Sex	Age	Comorbidities
1	M	38	None
2	M	19	None
3	M	37	None
4	F	51	None

## Data Availability

The data presented in this study are available on request from the corresponding author.

## Supplementary Materials

The following are available online at https://www.mdpi.com/article/10.3390/antibiotics10091087/s1, Figure S1: Cytotoxicity dose time-curve of hTCs treated with 0 mg/mL vancomycin (saline only) comparing cells cultured in DMEM as a baseline of 100%, Figure S2: Microscopic representative images showing hTCs in culture.

## References

[1] Singh N. (2018). International Epidemiology of Anterior Cruciate Ligament Injuries. Orthop. Res. Online J..

[2] Parada S.A., Grassbaugh J.A., Devine J.G., Arrington E.D. (2009). Instrumentation-specific infection after anterior cruciate ligament reconstruction. Sports Health.

[3] Vadala G., Petrillo S., Buschini F., Papalia R., Denaro V. (2017). Posterolateral bundle reconstruction of the anterior cruciate ligament to restore rotational stability of the knee. J. Biol. Regul. Homeost Agents.

[4] Calvo R., Figueroa D., Anastasiadis Z., Vaisman A., Olid A., Gili F., Valderrama J.J., De La Fuente P. (2014). Septic arthritis in ACL reconstruction surgery with hamstring autografts. Eleven years of experience. Knee.

[5] Papalia R., Moro L., Franceschi F., Albo E., D’Adamio S., Di Martino A., Vadala G., Faldini C., Denaro V. (2013). Endothelial dysfunction and tendinopathy: How far have we come?. Musculoskelet. Surg..

[6] Schuster P., Schulz M., Immendoerfer M., Mayer P., Schlumberger M., Richter J. (2015). Septic Arthritis After Arthroscopic Anterior Cruciate Ligament Reconstruction: Evaluation of an Arthroscopic Graft-Retaining Treatment Protocol. Am. J. Sports Med..

[7] Stucken C., Garras D.N., Shaner J.L., Cohen S.B. (2013). Infections in anterior cruciate ligament reconstruction. Sports Health.

[8] Torres-Claramunt R., Pelfort X., Erquicia J., Gil-Gonzalez S., Gelber P.E., Puig L., Monllau J.C. (2013). Knee joint infection after ACL reconstruction: Prevalence, management and functional outcomes. Knee Surg. Sports Traumatol. Arthrosc..

[9] Wang C., Lee Y.H., Siebold R. (2014). Recommendations for the management of septic arthritis after ACL reconstruction. Knee Surg. Sports Traumatol. Arthrosc..

[10] Plante M.J., Li X., Scully G., Brown M.A., Busconi B.D., DeAngelis N.A. (2013). Evaluation of sterilization methods following contamination of hamstring autograft during anterior cruciate ligament reconstruction. Knee Surg. Sports Traumatol. Arthrosc..

[11] Schollin-Borg M., Michaelsson K., Rahme H. (2003). Presentation, outcome, and cause of septic arthritis after anterior cruciate ligament reconstruction: A case control study. Arthroscopy.

[12] Schuster P., Schlumberger M., Mayer P., Eichinger M., Gesslein M., Richter J. (2020). Soaking of autografts in vancomycin is highly effective in preventing postoperative septic arthritis after revision anterior cruciate ligament reconstruction. Knee Surg. Sports Traumatol. Arthrosc..

[13] Perez-Prieto D., Portillo M.E., Torres-Claramunt R., Pelfort X., Hinarejos P., Monllau J.C. (2018). Contamination occurs during ACL graft harvesting and manipulation, but it can be easily eradicated. Knee Surg. Sports Traumatol. Arthrosc..

[14] Badran M.A., Moemen D.M. (2016). Hamstring graft bacterial contamination during anterior cruciate ligament reconstruction: Clinical and microbiological study. Int. Orthop..

[15] Vertullo C.J., Quick M., Jones A., Grayson J.E. (2012). A surgical technique using presoaked vancomycin hamstring grafts to decrease the risk of infection after anterior cruciate ligament reconstruction. Arthroscopy.

[16] Eriksson K., Karlsson J. (2016). Local vancomycin in ACL reconstruction: A modern rationale (2016) for morbidity prevention and patient safety. Knee Surg. Sports Traumatol. Arthrosc..

[17] Phegan M., Grayson J.E., Vertullo C.J. (2016). No infections in 1300 anterior cruciate ligament reconstructions with vancomycin pre-soaking of hamstring grafts. Knee Surg. Sports Traumatol. Arthrosc..

[18] Perez-Prieto D., Torres-Claramunt R., Gelber P.E., Shehata T.M.A., Pelfort X., Monllau J.C. (2016). Autograft soaking in vancomycin reduces the risk of infection after anterior cruciate ligament reconstruction. Knee Surg. Sports Traumatol. Arthrosc..

[19] Kirst H.A., Thompson D.G., Nicas T.I. (1998). Historical yearly usage of vancomycin. Antimicrob. Agents Chemother..

[20] Brophy R.H., Wright R.W., Huston L.J., Nwosu S.K., Group M.K., Spindler K.P. (2015). Factors associated with infection following anterior cruciate ligament reconstruction. J. Bone Jt. Surg. Am..

[21] Antoci V., Adams C.S., Hickok N.J., Shapiro I.M., Parvizi J. (2007). Antibiotics for local delivery systems cause skeletal cell toxicity in vitro. Clin. Orthop. Relat. Res..

[22] Hantes M.E., Basdekis G.K., Varitimidis S.E., Giotikas D., Petinaki E., Malizos K.N. (2008). Autograft contamination during preparation for anterior cruciate ligament reconstruction. J. Bone Jt. Surg. Am..

[23] Grayson J.E., Grant G.D., Dukie S., Vertullo C.J. (2011). The in vitro elution characteristics of vancomycin from tendons. Clin. Orthop. Relat. Res..

[24] Andrews J.M. (2001). Determination of minimum inhibitory concentrations. J. Antimicrob. Chemother..

[25] Offerhaus C., Balke M., Hente J., Gehling M., Blendl S., Hoher J. (2019). Vancomycin pre-soaking of the graft reduces postoperative infection rate without increasing risk of graft failure and arthrofibrosis in ACL reconstruction. Knee Surg. Sports Traumatol. Arthrosc..

[26] Naendrup J.H., Marche B., de Sa D., Koenen P., Otchwemah R., Wafaisade A., Pfeiffer T.R. (2020). Vancomycin-soaking of the graft reduces the incidence of septic arthritis following ACL reconstruction: Results of a systematic review and meta-analysis. Knee Surg. Sports Traumatol. Arthrosc..

[27] Aslantürk O.S. (2018). In Vitro Cytotoxicity and Cell Viability Assays: Principles, Advantages, and Disadvantages. Genotoxicity-A Predict. Risk Our Actual World.

[28] Riss T.L., Moravec R.A., Niles A.L., Duellman S., Benink H.A., Worzella T.J., Minor L., Sittampalam G.S., Grossman A., Brimacombe K., Arkin M., Auld D., Austin C.P., Baell J., Bejcek B., Caaveiro J.M.M., Chung T.D.Y. (2004). Cell Viability Assays. Assay Guidance Manual.

[29] Shaw K.A., Eichinger J.K., Nadig N., Parada S.A. (2018). In Vitro Effect of Vancomycin on the Viability of Articular Chondrocytes. J. Orthop. Trauma.

[30] Yazdi H.R., Jamei Moayedi R., Shokrgozar M.A., Dehghan M.M., Mokhtari T. (2014). Evaluation of delayed effect of intra-articular injection of cefazolin, gentamicin and vancomycin on articular cartilage: An experimental study in rabbit. J. Res. Orthop. Sci..

[31] Rathbone C.R., Cross J.D., Brown K.V., Murray C.K., Wenke J.C. (2011). Effect of various concentrations of antibiotics on osteogenic cell viability and activity. J. Orthop. Res..

[32] Edin M.L., Miclau T., Lester G.E., Lindsey R.W., Dahners L.E. (1996). Effect of cefazolin and vancomycin on osteoblasts in vitro. Clin. Orthop. Relat. Res..

[33] Chiu C.H., Lei K.F., Chan Y.S., Ueng S.W.N., Chen A.C. (2019). Real-time detection of antibiotics cytotoxicity in rabbit periosteal cells using microfluidic devices with comparison to conventional culture assays. BMC Musculoskelet. Disord..

[34] Liu J.X., Bravo D., Buza J., Kirsch T., Kennedy O., Rokito A., Zuckerman J.D., Virk M.S. (2018). Topical vancomycin and its effect on survival and migration of osteoblasts, fibroblasts, and myoblasts: An in vitro study. J. Orthop..

[35] Schuttler K.F., Scharm A., Stein T., Heyse T.J., Lohoff M., Sommer F., Spiess-Naumann A., Efe T. (2019). Biomechanical and microbiological effects of local vancomycin in anterior cruciate ligament (ACL) reconstruction: A porcine tendon model. Arch. Orthop. Trauma Surg..

[36] Pouzaud F., Bernard-Beaubois K., Thevenin M., Warnet J.M., Hayem G., Rat P. (2004). In vitro discrimination of fluoroquinolones toxicity on tendon cells: Involvement of oxidative stress. J. Pharmacol. Exp. Ther..

