# Supplementary material for: Does Vancomycin Wrapping in Anterior Cruciate Ligament Reconstruction Affect Tenocyte Activity In Vitro?
Source: Antibiotics (Basel). 2021 Sep 8;10(9):1087. doi: 10.3390/antibiotics10091087 (PMC8466500; doi:10.3390/antibiotics10091087)
Supplement: Supplementary file 1 [file antibiotics-10-01087-s001.zip › antibiotics-1328835-supplementary-done.pdf]

antibiotics

MDPI

Article

Does Vancomycin Wrapping in Anterior Cruciate Ligament Reconstruction Affect Tenocyte Activity in Vitro?

Rocco Papalia †, Claudia Cicione †, Fabrizio Russo, Luca Ambrosio, Giuseppina Di Giacomo, Gianluca Vadalà * and Vincenzo Denaro

Laboratory of Regenerative Orthopaedics, Department of Orthopaedic and Trauma Surgery, Campus BioMedico University of Rome, Rome, 00128, Italy; r.papalia@unicampus.it (R.P.); c.cicione@unicampus.it (C.C.); fabrizio.russo@unicampus.it (F.R.); l.ambrosio@unicampus.it (L.A.); g.digiacomo@unicampus.it (G.D.G.); denaro@unicampus.it (V.D.)

* Correspondence: g.vadala@unicampus.it

† The authors equally contributed to the work

Supplementary Materials

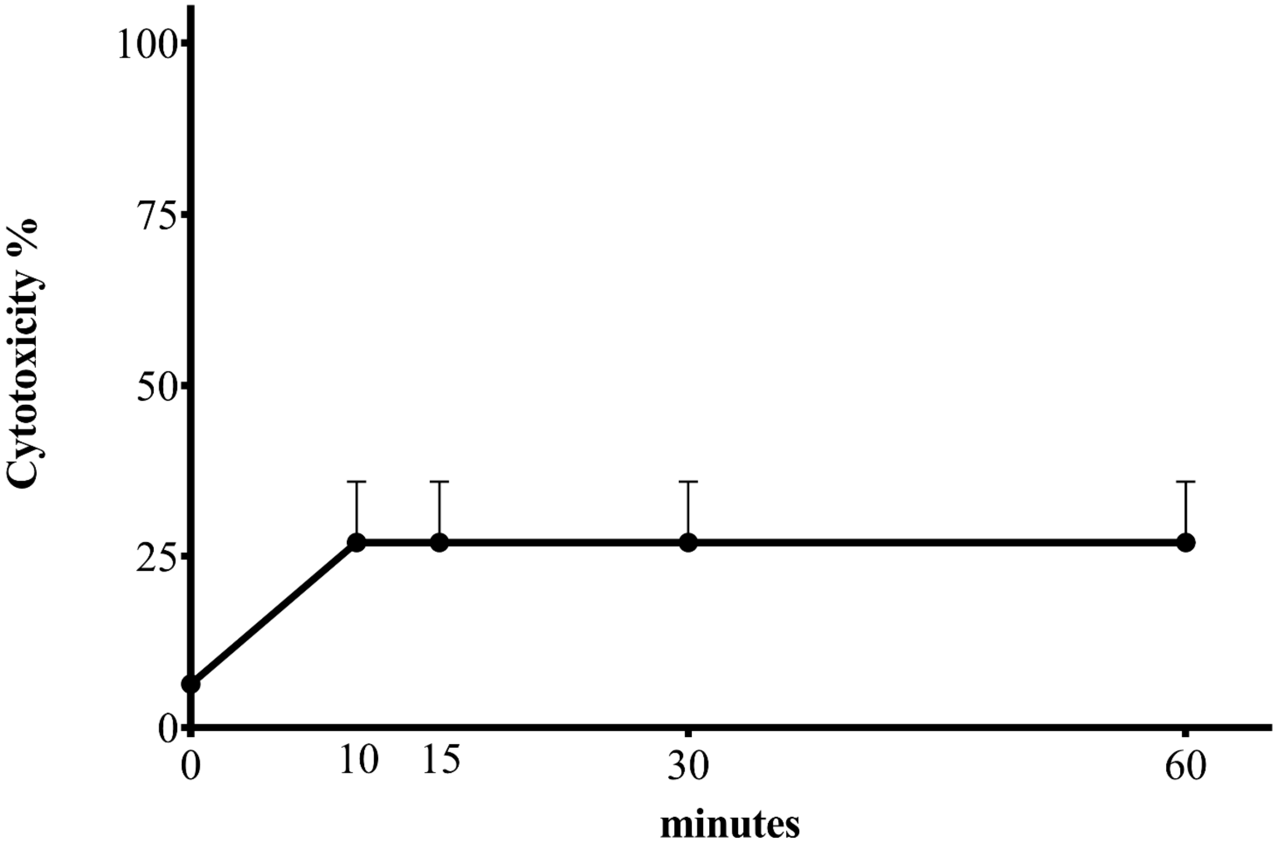

Figure S1. Cytotoxicity dose time-curve of hTCs treated with 0 mg/mL vancomycin (saline only) comparing cells cultured in DMEM as a baseline of 100%.

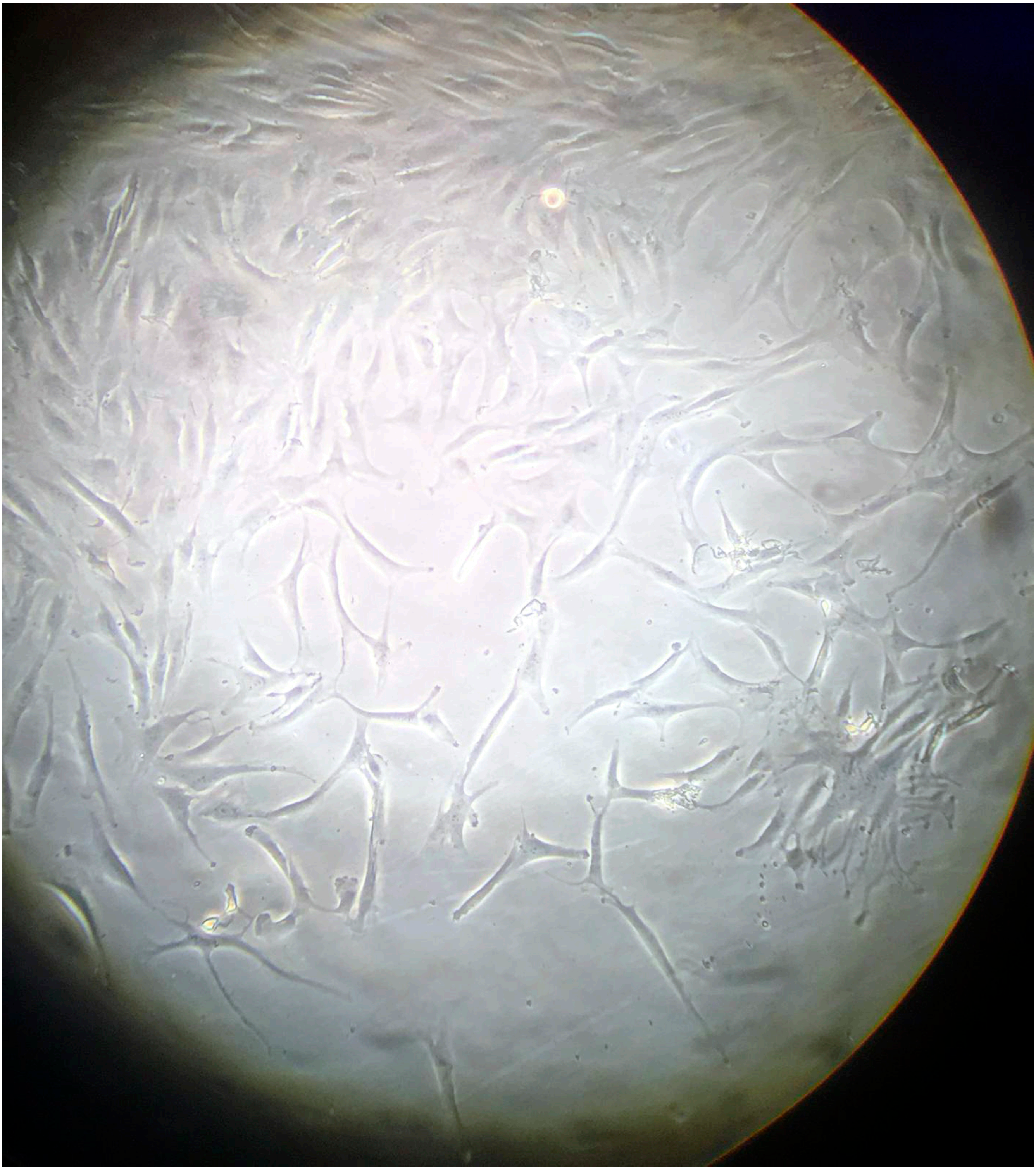

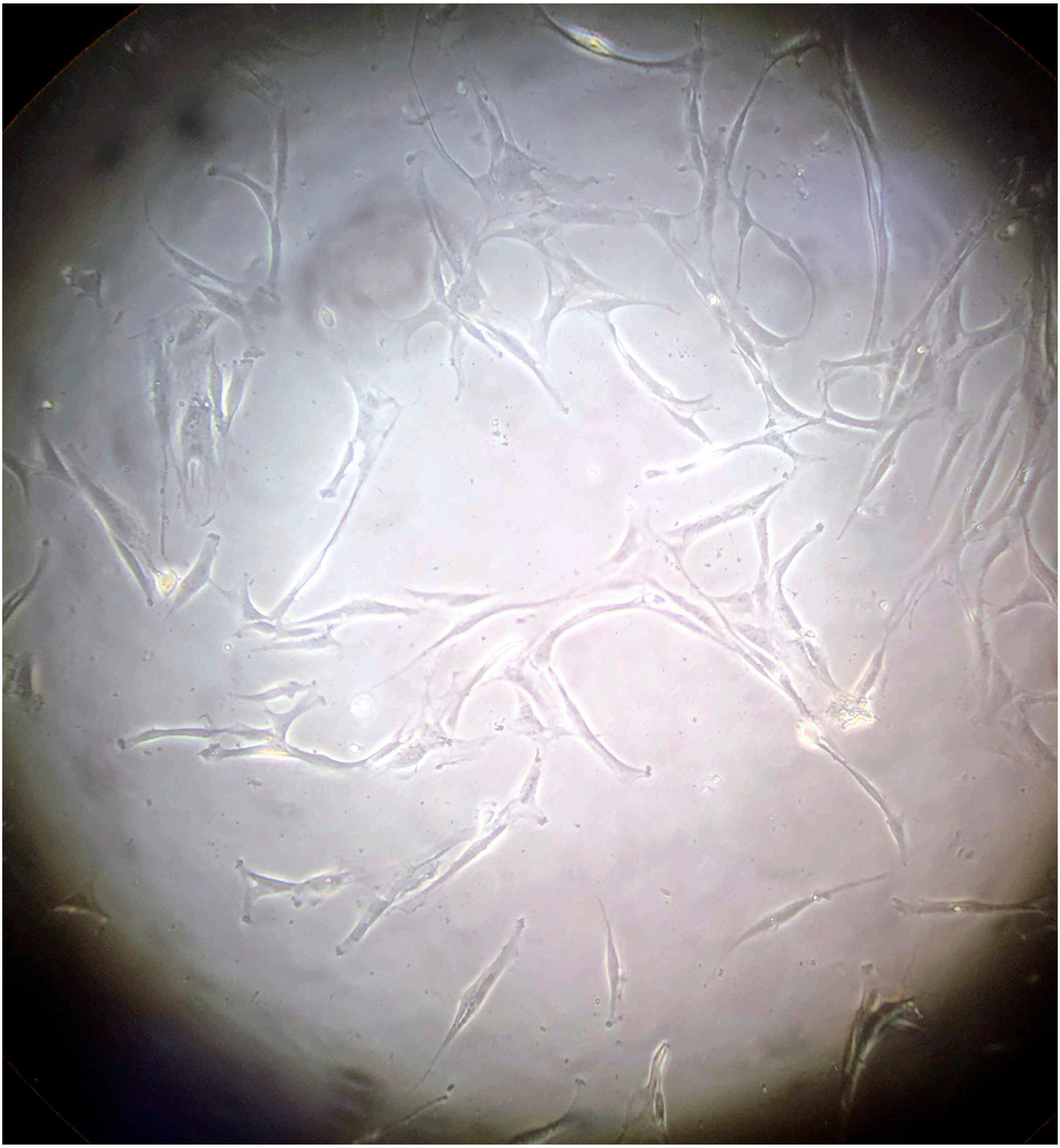

Figure S2. Microscopic representative images showing hTCs in culture.
